# The Neural Correlates of Food Preference among Music Kinds

**DOI:** 10.3390/foods13071127

**Published:** 2024-04-08

**Authors:** Yuanluo Jing, Ziyuan Xu, Yazhi Pang, Xiaolin Liu, Jia Zhao, Yong Liu

**Affiliations:** 1 Faculty of Psychology, Southwest University, Chongqing 400715, China; jingyl0209@163.com (Y.J.); xiaoshi.pang.20@alumni.ucl.ac.uk (Y.P.); jiazhao@swu.edu.cn (J.Z.); 2 Division of Psychology and Language Sciences, University College London, London WC1H 0AP, UK; ziyuanxu2001bella@gmail.com; 3 School of Music, Southwest University, Chongqing 400715, China; liumusicpsy@163.com; 4 Key Laboratory of Cognition and Personality (Ministry of Education), Southwest University, Chongqing 400715, China

**Keywords:** music kinds, taste, calorie, N2, P2

## Abstract

The calorie and taste choices of food have been shown to be related to the external environment, including music. Previous studies have mostly focused on manipulating basic auditory parameters, with few scholars exploring the impact of complex musical parameters on food selection. This study explored the effects of different kinds of music (classical, rock, jazz, and hip-hop) on food liking based on the calories (high and low) and taste (sweet and salty) using event-related potentials (ERPs). Twenty-four participants (8 males, 16 females) were recruited from Southwest University, China to participate in the food liking task using a Likert seven-point rating and simultaneously recording EEG signals (N2, P2, N3, and LPC). This study used repeated-measures analyses of covariances and found that the score of the high-calorie foods was greater than that of the low-calorie foods. Additionally, results revealed that the score in classical music was greatest for sweet foods, while there was no difference among music kinds in the salty foods. The ERP results showed that P2 amplitudes were greater for sweet foods than those for the salty foods. N2 amplitudes for the salty foods were greater than those for the sweet foods during rock music; in addition, N2 amplitudes during hip-hop music were greatest for sweet foods. However, N2 amplitudes during rock music were the greatest for salty foods. The results also revealed that N2 amplitudes during hip-hop music were greater than those during jazz music. This study provides unique operational insights for businesses.

## 1. Introduction

The interplay between music and human cognition has been a subject of extensive research, delving into the profound ways in which auditory stimuli can shape emotional states [1], cognitive processes [2], and even perceptual experiences [3]. While the influence of music on mood [1], memory [4], and attention [5] has been well-documented, the exploration of its impact on the evaluation of the sensory domain, specifically the gustatory experience of food, represents an emerging and fascinating area of study [6,7]. The aim of this research is to unravel the intricate connections between different kinds of music and food liking with varying calorie content and taste profiles.

Food is paramount to people, serving as the foundation and prerequisite for individual survival. After meeting survival requirements, individuals develop preferences for certain foods. Researchers have found that inherent attributes of food (such as color [8], aroma [9], and taste [10]), individual traits [11], and eating environments [12] may all influence individuals’ food preference. Sound is a key component of eating environments, and the manifestation of different sounds may impact an individual’s food preference. Music is one form of sound expression and a relatively easily controllable element. For example, many restaurants aim to influence individuals’ food preference using music.

The association between music and eating behavior has been confirmed by many researchers [6,7,13]. Crossmodal coherence refers to the tendency of the cognitive system to consistently map specific dimensions or features of stimuli across different sensory channels or to match them in preferred orientations [14,15,16]. Based on this, a previous study found that classical music gave people a sweet and gentle auditory experience, which was reflected in individuals’ taste preference for sweet foods [17,18]. There are no clear boundaries between the senses, and the physiological and cognitive aspects of perceptual experience are interconnected [19,20,21]. Researchers have revealed the interrelationships between multiple senses, such as hearing and taste [20,21]. Building on this, researchers conducted a more systematic study on the relationship between music and dietary preferences. Most researches have focused on manipulating basic variables, such as volume, rhythm, and pitch height [22,23]. However, the complexity of music expression has a more diverse correlation with diet [7]. Music style can be regarded as a complex and stimulating property of music. Fiegel et al. [6] were the first to explore the influence of music style on food perception and acceptance and found that jazz could predict participants’ preference for high-calorie emotional foods, such as milk chocolate, compared to rap and rock music. Additionally, another recent study found that the influence of background music on participants’ food preference depended on the interaction between music style, food health, and food taste [7]. Listening to jazz and classical music increased people’s preference for healthy salty foods, such as vegetable sandwiches [7].

The taste of food is an important factor that individuals consider when eating, and it is related to people’s food choices. A previous study has shown that brass instruments correspond to perceived bitterness, while the piano sound corresponds to perceived sweetness [24]. Wang et al.’s [25] experiment only used basic vocabulary such as sour, sweet, bitter, and salty as carriers of “taste”. Participants perceived “salty”, “sour”, and “sweet” music in correspondence with respective tastes, although “bitter” music was an exception. Peng Li et al. [26] conducted experiments using eye-tracking techniques and revealed that “sweet” or “salty” music caused participants to pay more attention to the corresponding sweet or salty foods, respectively. Guedes et al. [27] extended previous researches on music genres and established a flavor music database. In the modern field of diet, individuals pursue healthier dietary habits. Generally speaking, low-calorie and low-fat foods correspond to “health,” while high-calorie and fatty foods represent “unhealthy” [13]. Peng Li et al. [13] created original “healthy” music (jazz music with piano instruments) and “unhealthy” music (disharmonious guitar melodies with brass chord progression) based on the results of pre-experiments. Whether analyzing behavioral outcomes based on food selections or eye movement outcomes based on gaze patterns, the study revealed that “healthy” music matched healthy food, and “unhealthy” music matched unhealthy food [26]. It is worth noting that a study exploring the sound–taste connection found that, although different music had a significant effect on sweetness or saltiness, the dependence of choosing high-calorie desserts on background sound was smaller than that of low-calorie desserts [28].

Apart from behavioral and eye-tracking experiments, researchers are also exploring the relationship between music and diet from the perspective of EEG. Music, as a potent emotional and cognitive stimulus, possesses the ability to evoke a wide array of emotions and alter the neural substrates that underlie these emotional experiences [29]. Therefore, it may alter an individual’s brainwave. The N2 is a negatively deflected and stimulus-responsive ERP (event-related potential) [30]. When more conflicts are detected, N2 will exhibit a greater amplitude [31,32]. A previous study has shown that individuals who receive music training exhibit larger N2 amplitudes [33]. Even brief music listening can have an impact on N2. A study has shown that calm music and happy music can regulate negative emotions and reduce N2 amplitude [34]. Meanwhile, one study revealed that inhibitory responses to high-calorie stimuli elicited larger N2 amplitudes, while inhibitory responses to low-calorie stimuli elicited smaller N2 amplitudes [35]. In addition to the calorie content of food, taste may also cause changes in N2. The first successful taste ERP record was reported half a century ago, but since then, there have been few publications on taste ERP [36,37]. A study has shown that the posterior lateral orbitofrontal cortex, primary somatosensory cortex and ipsilateral insular-opercular cortex have been shown to receive fascicles from the ventroposteromedial nucleus of the thalamus [38]. It seems that ERP, as an indicator for monitoring real-time brain responses, can also be used to monitor taste responses [39]. However, there are still very few ERP experiments on taste perception at present.

In addition to focusing on N2, P2 is also an important indicator in ERP. The increase in P2 amplitude is an indicator related to auditory learning [40]. In a research report, P2 amplitude increased after various types of auditory training [39,41]. Lee [42] conducted an experiment on 60 participants to assess the accuracy of visually presented sentences while listening to music with lyrical content, music without lyrical content, or silence. The results indicated that P2 under silent conditions was larger than under music conditions with lyrics. In addition, the variation of P2 depends on the stimulus value [43]. A previous study asked participants to perform food no-go and flower no-go tasks. In both tasks, the P2 amplitude of overweight individuals was higher than that of normal-weight individuals [44]. A study found that an increase in P2 amplitude in obese participants indicated an increased bias towards food-related stimuli [45], but food intake led to a decrease in P2 amplitude in restricted-diet participants [46]. Stockburger [47] required participants to passively watch a series of images (both food and non-food), with hungry participants exhibiting higher P2 amplitudes than full participants.

N3 is a negative potential that occurs 250–350 ms after the appearance of the stimulus [48] and is related to the emotional processing of visual stimuli [49]. It reflects the early perception process of emotional stimuli [49]. LPC is a positive potential that occurs approximately 600–1000 ms after stimulation [50], reflecting the regulation of emotional stimuli [51]. The increase in LPC amplitude can be explained by the application of more cognitive resources to emotional processing [52]. The amplitude of LPC induced by negative images is greater than the amplitudes induced by positive and neutral images [53]. Liu [34] divided the participants into three groups and used music to induce three emotions: calm, happy, and sad. The results showed that the sad-music group exhibited larger N3 and LPC amplitudes.

Previous studies have mostly focused on the impact of basic music parameters on food preference [13,22,23], and the classification of food preference has focused on either calorie or taste alone [22,23,27]. The research divides music kinds into four categories (classical, rock, jazz, and hip-hop) and combines food taste and calories as well as ERP to make the experiment more realistic and diverse. It uses music kinds as the independent variables, and the food liking scores and P2, N2, N3, and LPC amplitude changes as the dependent variables to clarify individual food liking preferences after four different music listening. In addition to revealing behavioral outcomes, ERP results demonstrate changes in the brain’s electrical signals, exploring the influence of different music kinds on an individual’s food liking. Based on the above literature, we propose a hypothesis that different music styles can lead to preference for different dietary calories and tastes, and the ERP components will exhibit differences as well. We speculate that individuals prefer high-calorie foods across music kinds. More specifically, classical music leads to individuals scoring higher in food liking tasks, accompanied by an increase in P2 and N2, and a decrease in N3 and LPC amplitudes.

## 2. Methods

### 2.1. Participants

Posting recruitment information online, twenty-four college student participants (8 males, 16 females; M_age_ = 20.58, SD_age_ = 1.26) were recruited from Southwest University in China, covering different grades and majors. All participants reported having no history of major psychological disorders, normal weight (M_BMI_ = 21.07, SD_BMI_ = 2.32), and normal or corrected-to-normal vision. Participants reported no use of chronic or acute medications. In addition, participants self-reported any picky eating behaviors, and those who reported such behaviors were excluded from the study. We spent two weeks collecting all formal experimental information from the participants. All participants provided written consent prior to completing the experiment. After the experiment completed, all participants received experimental compensation. We used GPower 3.1 for post hoc power analyses, and the results showed that when the effect size was set to 0.2, our sample size (n = 24) had a power (1 − β) of 0.96. The present study was approved by the Southwest University Ethics Committee (IRB No. H22020).

### 2.2. Hunger and Desire to Eat

All participants rated their hunger and desire to eat on a 100-mm visual analog scale (VAS) from “not at all” to “very high”.

### 2.3. Stimuli and Food Liking Task

The music used in the present study was from previous studies [6,7]. We selected 20 music segments, 5 segments each for the classical, jazz, hip-hop, and rock music kinds. Each music segment was presented for 30 s. The study used the QQ music software (v:11.0.0.10) to download the original music and used Permute 3.0 to convert the format of the audio and cut out the required segments based on the study conducted by Motoki et al. [7].

The food images, based on their calories (high or low calorie) and taste (sweet or salty), were selected from the Chinese food-image database conducted by our team [54]. In the present study, four kinds of food images were high-calorie and sweet foods, high-calorie and salty foods, low-calorie and sweet foods, and low-calorie and salty foods. Each kind had 40 images; the total of food images was 160.

**Figure 1:**
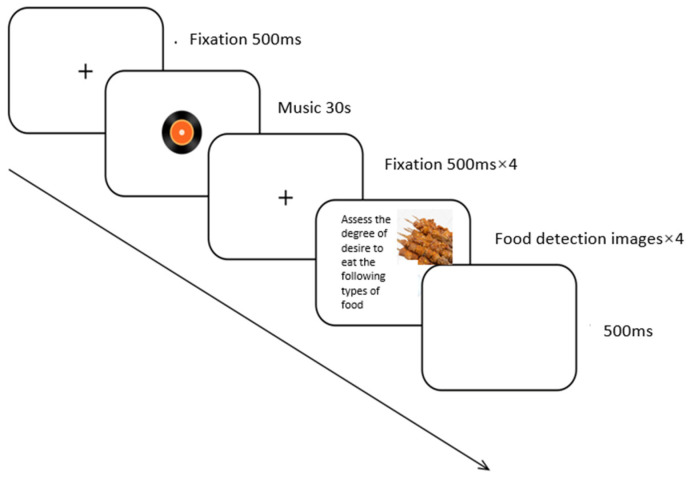
The food liking task used in the study.

During the food liking task (Figure 1), the fixation appeared on the screen for 500 ms. Then, the music segments were displayed for 30,000 ms. Before the food images were displayed on the screen, a second fixation appeared for 500 ms. In the task, all participants were asked to report food liking using a Likert 7-point rating (1 = not at all, 7 = very much). Following this, a white screen would appear for 500 ms after the stimulus screen. During the task, after listening to each music segment, the participant rated the liking of the four kinds of food images. Therefore, music segments were presented randomly, while food images were presented pseudo-random to avoid presenting the same kind of food images repeatedly.

### 2.4. Procedure

Participants were asked to abstain from consuming any substances except for water for at least 4 h leading up to the experiment for standardization purposes [55,56]. After providing written consent, participants completed the hunger rating and desire to eat. Then, participants performed the food liking task, while EEG data were recorded.

### 2.5. EEG Recording and Analysis

Brain electrical activities were recorded by a 64-channel wireless amplifier with a sampling frequency of 1000 Hz (NeuSen. W64, Neuracle, Changzhou, China). EEG data processing was performed with MATLAB R2022a using the EEGLAB toolbox [57]. EEG data was filtered with a bandpass finite impulse response (FIR) filter between 0.1 and 30 Hz. The left and the right mastoids were taken as re-reference sites. The continuous EEG data were divided into trials (−200 ms to 1000 ms) according to the different marks of stimuli. A baseline correction (−200 ms to 0 ms) was applied to each trial, and the quality of the EEG data was inspected trial by trial. Trials with abnormal fluctuations (too large or too frequent fluctuations) were eliminated, and trials with bad channels were corrected by averaging the amplitudes of their adjacent electrodes. The independent components analysis (ICA) method was applied to the EEG data to remove interference factors (e.g., eye movements, electrocardio, etc.) from the data. In the ICA results, components with EOG artifacts and head movement were removed after visual inspections [56]. The frontal site is representative of cognitive control and does not sit directly above the premotor and motor areas of the brain, which ensures that the quality of the data is not contaminated by the button-pressing motions [58]. Therefore, site Fz was selected for the data analysis. Based on the grand-averaged ERP activities, the ERPs and their time window were as follows: P2, 150–200 ms; N2, 200–260 ms; N3, 280–360 ms; and LPC, 500–1000 ms.

Repeated-measures analyses of covariances (ANCOVA, Greenhouse–Geisser-corrected) were conducted on the P2, N2, N3, and LPC amplitudes, with music kinds, calorie, and taste as within-subjects factors, and hunger and desire to eat as covariates. All analyses were conducted via SPSS 25.0. Based on the Greenhouse–Geisser method, *p*-values were computed for deviation in all analyses. Post hoc *t*-tests were conducted with Bonferroni correction for multiple pairwise comparisons.

## 3. Results

### 3.1. Behavioral Results

Repeated-measures analyses of covariances (ANCOVA, Greenhouse-Geisser-corrected), with music kinds, calorie, and taste as within-subjects factors, and hunger and desire to eat as covariates, revealed marginally significant main effects of calorie, F (1, 21) = 3.64, *p* = 0.07, partial η_p_^2^ = 0.15; the score of the high-calorie foods was greater than that of the low-calorie foods. The results also revealed an interaction of music kinds and taste, F (3, 63) = 3.00, *p* = 0.05, partial η_p_^2^ = 0.13; the follow-up simple analysis revealed that the score in classical music was greatest in the sweet foods, while there was no difference among music kinds in the salty foods. In addition, there was no difference between the sweet and salty foods during all music kinds, all *p* > 0.05. The details of behavioral data could be found in Table 1. We did not find an interaction between taste and calorie, F (1, 21) = 2.362, *p* = 0.139, partial η_p_^2^ = 0.101.

### 3.2. ERP Results (Figure 2)

#### 3.2.1. P2

The results on P2 amplitudes revealed a main effect of taste, F (1, 21) = 5.49, *p* = 0.029, partial η_p_^2^ = 0.21; P2 amplitudes in the sweet foods were greater than that in the salty foods. We did not find an interaction between taste and calorie, F (1, 21) = 0.162, *p* = 0.692, partial η_p_^2^ = 0.008.

#### 3.2.2. N2

The results on N2 revealed an interaction of music kinds and taste, F (3, 63) = 3.17, *p* = 0.047, partial η_p_^2^ = 0.13. N2 amplitudes in the salty foods were greater than that in the sweet foods during the rock music (*p* < 0.001); in addition, N2 amplitudes during the hip-hop music were greatest in the sweet foods, while N2 amplitudes during the rock music were greatest in the salty foods. The results also revealed a main effect of music kinds, F (3, 63) = 4.13, *p* = 0.017, partial η_p_^2^ = 0.16. A post hoc *t*-test showed that N2 amplitudes during the hip-hop music were greater than that during the jazz music, *p* = 0.004. We did not find an interaction between taste and calorie, F (1, 21) = 0.944, *p* = 0.342, partial η_p_^2^ = 0.043.

We did not find any difference in the N3 and LPC amplitudes; all *p* > 0.05.

**Figure 2:**
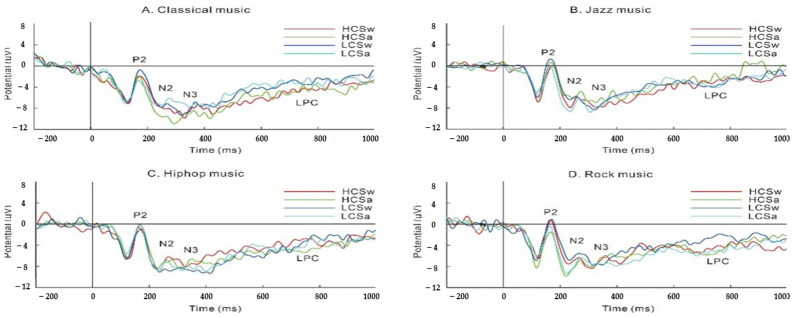
The ERP results during the food liking task. Note: HCSw, high-calorie and sweet foods; HCSa, high-calorie and salty foods; LCSw, low-calorie and sweet foods; LCSa, low-calorie and salty foods.

## 4. Discussion

The research divided music kinds into four categories (classical music, rock music, hip-hop music, and jazz music) and combined food taste and calorie as well as ERP. The results showed that individuals prefer to choose high-calorie foods. Classical music made individuals more willing to choose sweet foods than other kinds of music. The P2 amplitudes of sweet foods was greater than those of salty foods. During rock music, the N2 amplitudes of salty foods was greater than those of sweet foods. The N2 amplitudes during hip-hop music were highest in sweet foods, and during rock music, they were highest in salty foods. The amplitudes of N2 during hip-hop music were greater than those during jazz music. This study combined behavioral researches and ERP researches for conducting experiments and explored the impact of complex music parameters on food liking tasks and ERP.

The results showed that the score of high-calorie foods was higher than that of low-calorie foods, which was consistent with the previous literature [59,60,61,62,63]. Individuals have a natural preference for high-calorie foods, which promotes fat accumulation and better adapt to the environments [59,61]. In situations of hunger, the rewarding effect of high-calorie foods is more likely to induce people to choose them [60,62,63]. When performing food-preference tasks, individuals’ impulsive food decision-making behavior is often guided by the automatic associative process that occurs when perceiving food options [64]. When people look at food cues, taste is activated first [65], and the processing and integration of health attributes into food decision-making takes about 195 milliseconds longer than taste [66]. The food liking task requires a quick response. Therefore, high-calorie foods provide individuals with a strong sense of pleasure in taste, which can be more quickly attributed to food decision-making than health factors and allow individuals to consider taste factors more and choose high-calorie foods.

In the subsequent results, the results revealed a strong correlation between classical music and sweet foods, which confirmed previous findings. Kontukoski’s [18] research regarded some classical music as a model of sweet music. Knöferle [17] explained that this might be due to the higher tones, softer rhythms, and melodies of classical music, which were coordinated with sweetness in terms of auditory parameters. When people hear classical music, seeing photos of sweet foods can make them familiar, which may be one of the reasons why they prefer sweet foods.

North’s research confirmed that classical music helped individuals reduce their intake of salty foods [67]. The relationship between salty foods and music kinds was not found in the present study. In the dietary habits of Chongqing, China, salty foods align with people’s dietary habits. People have a relatively fixed choice and hobby of salty foods, so regardless of whether the music kinds changes, individuals may be more inclined to choose salty foods according to their own habits. This fixed choice of salty foods may also be related to the kinds of music one often listens to. The North experiment suggested that listening to a specific concert activated relevant memories in the brain, thereby affecting product selection [68]. Chinese people tend to eat more salty foods while listening to music, and this choice may also implicitly lead to a more consistent dietary pattern among individuals. The ethnic consistency of the correlation between music kinds and food has also been proven in the previous literature to affect food preference or choices [68,69,70] This study also found no difference between sweet and salty foods across all music kinds. Previous studies have shown that there is a bimodal split in preferences for sweetness and saltiness [71]. Among the Chinese public, popular music is more popular. These four kinds of music cannot universally influence the public’s preference for food. The choice of sweet and salty foods is not significantly correlated with these kinds of music.

With regard to ERP results, P2 may indicate a preference for stimuli [45]. The research results indicated that P2 amplitudes exhibited a higher amplitude in sweet foods. After several hours of starvation, individuals feel a more pronounced degree of hunger and lower blood sugar levels and are more inclined to choose foods that raise blood sugar levels faster to satisfy their hunger and increase their satiety [72]. In the theory of evolutionary psychology, the main goals of living organisms are survival and reproduction. People are more inclined to choose options that are beneficial for survival and reproduction [73]. This also explains the individuals’ tendency towards sweet foods in a state of hunger. In this study, the number of female participants was higher than that of male participants. Previous studies have shown that women have a stronger preference for sweet foods, which may also be one of the reasons why they choose more sweet foods [74].

N2 represents the ability of cognitive control [30]. During rock music, the amplitudes of N2 in salty foods were greater than that in sweet foods. Sweet foods can improve an individual’s mood [75]. Previous studies have shown that specific styles of music provide emotional guidelines, such as classical music, movie soundtracks, and Latin music, which are endowed with different emotional experiences [27]. Rentfrow and Gosling [76] classified different kinds of music into four dimensions. For example, the dimension of “reflection and complexity” included blues, classical, jazz, and folk; the dimensions of “intensity and rebellion” included rock, alternative rap, and heavy metal music. The dimensions of “optimism” and tradition included country music, film and television soundtracks, religious music, and pop music; the dimension of “vitality and rhythm” included rap music, soul/funk music, and electronic/dance music. The intensity and rebellion of rock music, with its more passionate and intense music, can also excite people and make them more impulsive [77]. Impulsiveness is related to sweet foods [78], which causes more sweet foods intake. This shows that sweet foods match rock music, and rock music is more likely to awaken the craving for sweet foods. Those who choose salty foods allocated greater cognitive control to encourage themselves to choose salty foods.

Hip-hop music originates from the African-American community and has a strong sense of rhythm. Due to the limited melodies, it is difficult to cause individual emotional fluctuations, especially in China where hip-hop music belongs to niche music and has difficulty resonating with the public. Hip-hop music cannot effectively influence food choices. Therefore, in the context of hip-hop music, individuals tend to choose salty foods that appear more frequently and are more accustomed to in daily life. When choosing sweet foods, more cognitive resources are required. Fiegel et al. [6] found that people reported higher levels of emotional pleasure when eating while listening to jazz, and compared to hip-hop music, jazz could better enhance individual pleasure. A study has shown that happy music can regulate negative emotions and reduce N2 amplitudes [34]. This also explains why the amplitudes of N2 during hip-hop music were greater than those during jazz music. Indeed, different music can also give people different emotional experiences; for example, techno dance and Latin American music often bring energetic emotions to the audience. The differences between different types of emotions induced by music may lead to approach-avoidance behavior [79], which, in turn, leads to differences in the individual perception of food under different kinds of music [6]. N3 and LPC, as emotional indicators, have no significant effect, possibly because individuals do not allocate attention to emotions and only unconsciously experience emotional changes.

## 5. Limitation

There are limitations associated with the present study. First, this study did not measure individual preference for different kinds of music before the experiment, and the degree of individual preference for different kinds of music may affect the results. Future research can measure individual preference for music before conducting experiments. Second, this task was conducted only after listening to music and cannot fully reflect the impact of music on individual food liking at that time. Future research can attempt to perform tasks while listening to music. Third, we conducted repeated-measures analyses of covariances, and causality cannot be inferred. Further study can explore causality through long-term music-training interventions.

## 6. Conclusions

In summary, this study demonstrates that different kinds of music can indeed influence the intake of food calorie and taste. Classical music is associated with sweet foods, and the P2 amplitudes of sweet foods are greater than those of salty foods, which may be because classical music harmonizes with sweetness. Under rock music, the N2 amplitudes of salty foods are greater than those of sweet foods. This is because the impulsiveness of rock music triggers more sweet foods, resulting in less salty-food-eating behaviors. In hip-hop music, N2 amplitudes are highest among sweet foods, while in rock music, N2 amplitudes are highest among salty foods. Furthermore, the amplitudes of N2 during hip-hop music are greater than those during jazz music. The potential mechanism in this study for correlations between different kinds of music and food liking is the change in ERP components. The study establishes a correlation between four kinds of music and various dietary choices, providing unique insights for businesses and a foundation for future research. This conclusion can be extended to the general population of college students.

## Figures and Tables

**Table 1:** Descriptive statistics (M ± SD) for all participants in the food liking task.

Music	High-Sweet	High-Salty	Low-Sweet	Low-Salty	Mean
Classical	5.02 (1.13)	5.26 (0.83)	4.87 (0.86)	4.34 (1.10)	4.87 (0.98) a
Jazz	4.89 (0.99)	5.38 (0.76)	4.67 (0.95)	4.27 (1.04)	4.80 (0.94) a
Hip-hop	4.73 (1.21)	5.38 (0.78)	4.64 (0.88)	4.20 (1.06)	4.73 (0.98) b
Rock	4.83 (1.18)	5.41 (0.78)	4.70 (0.98)	4.05 (1.07)	4.75 (1.00) b
Mean	4.87 (1.13) b	5.36 (0.79) a	4.72 (0.92) b	4.22 (1.07) c	

Note. Different subscripts indicate significant mean differences among the conditions (music kinds and foods) (*p* < 0.05).

## Data Availability

The original contributions presented in the study are included in the article, further inquiries can be directed to the corresponding authors.
